# What are the learning outcomes of a short postgraduate training course in dermatology for primary care doctors?

**DOI:** 10.1186/1472-6920-11-20

**Published:** 2011-05-16

**Authors:** TP Lam, CK Yeung, KF Lam

**Affiliations:** 1 Department of Family Medicine and Primary Care, The University of Hong Kong, Hong Kong; 2 Division of Dermatology, Department of Medicine, The University of Hong Kong, Hong Kong; 3 Department of Statistics and Actuarial Science, The University of Hong Kong, Hong Kong

**Keywords:** continuing medical education, dermatology, learning outcomes, postgraduate training, primary care doctors

## Abstract

**Background:**

There are increasing expectations on primary care doctors to shoulder a bigger share of care for patients with common dermatological problems in the community. This study examined the learning outcomes of a short postgraduate course in dermatology for primary care doctors.

**Methods:**

A self-reported questionnaire developed by the research team was sent to the Course graduates. A retrospective design was adopted to compare their clinical practice characteristics before and after the Course. Differences in the ratings were analysed using the nonparametric Wilcoxon signed rank test to evaluate the effectiveness of the Course in various aspects.

**Results:**

Sixty-nine graduates replied with a response rate of 42.9% (69/161). Most were confident of diagnosing (91.2%) and managing (88.4%) common dermatological problems after the Course, compared to 61.8% and 58.0% respectively before the Course. Most had also modified their approach and increased their attention to patients with dermatological problems. The number of patients with dermatological problems seen by the graduates per day showed significant increase after the Course, while the average percentage of referrals to dermatologists dropped from 31.9% to 23.5%. The proportion of graduates interested in following up patients with chronic dermatological problems increased from 60.3% to 77.9%.

**Conclusions:**

Graduates of the Course reported improved confidence, attitudes and skills in treating common dermatological problems. They also reported to handle more patients with common dermatological problems in their practice and refer fewer patients.

## Background

Studies show that 8-15% of primary care consultations involve a dermatological problem [1-3]. There are increasing expectations on primary care doctors to shoulder a bigger share of care for patients with common dermatological problems in the community [4,5]. However, some primary care doctors were inadequately trained to meet these expectations [6,7]. Studies indicated that undergraduate training in dermatology is insufficient in many countries [8,9], and the need for vocational training and continuing medical education in dermatology has been emphasized [10-13].

In recent years, there is a global trend to improve the quality of primary care doctors in terms of their medical knowledge and practice through postgraduate studies [14,15]. Studies showed that primary care doctors exhibited improvements in clinical practices, patient care and lifelong learning interest after completing postgraduate courses [16,17]. There is however little information available in the literature on the possible effect of postgraduate training on improving the skills and confidence of primary care doctors in the care of patients with common dermatological problems as well as the rate of referrals to dermatologists [18,19].

In Hong Kong, undergraduate dermatology and venereology training in medical schools consists of series of systemic lectures, tutorials, bedside and outpatient teaching during the clinical years. There are about 24 hours of undergraduate teaching in total. This is broadly similar to many medical schools around the world. A Certificate Course in Clinical Dermatology (CCCD) specially designed for primary care doctors was launched in 2007 by the Department of Family Medicine and Primary care (formerly Family Medicine Unit) and Division of Dermatology, Department of Medicine of The University of Hong Kong. The Course was the outcome of a close collaborative effort by both family physicians and dermatologists after detailed discussions on the needs of doctors in primary care setting and the method of delivery. It was also developed in response to the community needs in Hong Kong as there are currently only 81 specialist dermatologists practising in Hong Kong serving a population of 7 million. The Course comprised of 20 seminars conducted by specialists in dermatology, plastic surgery, paediatrics and microbiology using a problem-oriented approach. The speakers would present a clinical problem with a clinical picture and asked students' feedback when a new topic was introduced. It was designed to provide a comprehensive review of the dermatological problems commonly seen at primary care setting and to update the participants in the diagnosis and management of these conditions. There were 10 weekly 2-hour sessions, with 2 seminar topics for each session. A total attendance of at least 80% of the sessions was required for course completion. The study topics are shown in Table 1.

**Table 1:** The study topics of the training programme

Study topics
Management approach to eczema/dermatitis Acne and rosacea Psoriasis and other scaling eruptions Diagnostic algorithms for common dermatoses Common cutaneous infections Common facial dermatoses Allergy and urticaria Geriatric skin disorders Paediatric skin diseases Common scalp, hair and nail disorders Genital dermatoses Sexually transmissible diseases Skin and systemic diseases Cosmetic dermatology Common dermatological therapy Benign and malignant skin tumours Practical guide in skin surgery Drug eruptions

Over 190 doctors, with the great majority being primary care doctors, have been trained in this Course since 2007. It was expected that the Course would have significant impact on these doctors, including improvements in clinical skills, attitude, confidence, and changes in number of patients handled in relation to dermatological problems and referral rates. Our study aimed to investigate and report these changes.

## Methods

### Study design

A self-reported questionnaire for the Course graduates was developed based on the review of relevant literature and comments from research team members. Likert scale questions and open-ended questions were included to obtain both quantitative and qualitative data. A retrospective design was adopted to compare the clinical practice characteristics of the graduates before and after the Course. The questionnaire was pilot-tested in July 2008 and finalized the following month. A lucky draw (three prizes of US$60 book coupon each) was carried out amongst all those who had completed and returned their questionnaires as an incentive measure. Ethics approval was obtained from the local Institutional Review Board.

### Data collection

Copies of the questionnaire, each enclosed with an invitation letter and a pre-paid return envelope, were sent to all graduates of CCCD of year 2007. The questionnaire itself was anonymous but coded with a reference number to identify the respondent for the lucky draw and for subsequent rounds of reminders. The code was known to a research assistant only and not available to members of the research team. A total of 163 questionnaires were sent to the CCCD graduates in August 2008 i.e. at least 8 months after completing the Course to avoid premature comments from fresh graduates. Non-respondents were sent up to two reminders between September and December 2008. To improve the response rate, doctors who had not responded were contacted by telephone after the first reminder.

### Data Analysis

Quantitative analysis was carried out using the statistical software SPSS version 17.0. The measurements were mainly made in ordinal scale, statistical inference via the nonparametric Wilcoxon signed rank test was used to determine if there were significant changes in the median of the differences in the responses before and after taking the Course. A negative value in the median of the differences was an indication that the respondents tended more on the agreed side or a higher number reported after taking the Course. In our study, there were various items to be tested. Improvements (increase) were assumed in cases regarding confidence and attitude, while a change in referrals rates (either increase or decrease) was expected. Two-sided tests were used throughout this study whenever appropriate. A p-value < 0.05 was considered statistically significant. In the sequel, we shall simply quote only the *p*-values and draw conclusions instead of going through the description of testing the null hypothesis that the median is zero indicating no change in the responses after taking the Course. The description in each case would emphasize on the pattern of the differences when the null hypothesis is rejected. Furthermore, the Kappa statistics and the Bowker's test of symmetry were used to measure the degree of agreement and test for symmetry between the responses before and after the Course. Small values of Kappa and p-value based on the Bowker's test are associated with significant changes in the responses before and after taking the Course.

The qualitative responses were analyzed with a thematic approach and grouped into common themes independently by one of the authors, TPL and a research assistant who were both experienced in qualitative research. The consistency between the two entries was checked.

## Results

### Respondents' characteristics

Out of the 163 questionnaires sent to the graduates, 2 postal addresses were invalid. We received 69 replies after three rounds of invitation, with a response rate of 42.9% (69/161). Of the respondents, 65.7% were male and 34.3% female, 66.2% were in private service and 33.8% were in public service. Majority (82.5%) of the respondents were primary care doctors working in the community, and the remaining few were working in the specialties of internal medicine and paediatrics within the hospital setting. The mean (SD) years after graduation from medical school was 17.3 (10.70).

### The Main Learning Outcomes

Most (86.9%) respondents reported to have paid more attention to patients with dermatological problems after taking the Course. Similarly, 85.5% of the respondents had modified their approach to these patients and had increased their confidence in distinguishing different types of dermatological problems. However, only 36.7% of the respondents thought their career opportunities have been enhanced (Table 2).

**Table 2:** Learning outcomes of the Course (in descending order of combined frequencies of Likert scales 3 and 4)

**As a result of the course**,	strongly disagree	disagree	agree	strongly agree
	1	2	3	4
I have paid more attention to patients with dermatological problems	0 (0.0%)	9 (13.0%)	49 (71.0%)	11 (15.9%)
I have increased my confidence in distinguishing different types of dermatological problems	0 (0.0%)	10 (14.5%)	49 (71.0%)	10 (14.5%)
I have modified my approach to patients with dermatological problems	0 (0.0%)	10 (14.5%)	51 (73.9%)	8 (11.6%)
I have increased my interest in lifelong learning through additional training	1 (1.4%)	14 (20.3%)	44 (63.8%)	10 (14.5%)
I have increased my interest in pursuing other postgraduate studies	2 (2.9%)	20 (29.0%)	41 (59.4%)	6 (8.7%)
My career opportunities have enhanced	4 (5.9%)	39 (57.4%)	23 (33.8%)	2 (2.9%)

Likert scale from 1 (strongly disagree) to 4 (strongly agree); % on valid data across rows of the table

### Comparison of Reported Clinical Practice Before and After Taking the Course

The responses to this part and the results of Wilcoxon signed rank test are summarized in Tables 3 and 4.

**Table 3:** Reported changes in referrals and engagement in the care of patients with dermatological problems

	Pre-course	Post-course	Wilcoxon signed rank test
Percentage of patients with dermatological problems being referred to dermatologist* (n = 60)			
0 - 10	22 (36.7%)	30 (50.0%)	
11 - 20	10 (16.7%)	7 (11.7%)	
21 - 30	5 (8.4%)	7 (11.7%)	Z = 4.547, p < 0.001
31 - 40	1 (1.7%)	3 (5.0%)	
41 - 50	10 (16.7%)	8 (13.3%)	
Over 50	12 (20.0%)	5 (8.3%)	
Of all the referrals to dermatologist, percentage of patients being referred at first consultation* (n = 60)			
0	10 (16.7%)	11 (18.3%)	
1 - 20	22 (36.7%)	27 (45.0%)	
21 - 40	5 (8.3%)	7 (11.7%)	Z = 3.905, p < 0.001
41 - 60	9 (15.0%)	8 (13.3%)	
61 - 80	6 (10.0%)	2 (3.3%)	
81 - 100	8 (13.3%)	5 (8.3%)	
Number of patients with dermatological problems seen per week (*n *= 67)			
0 - 5	21 (31.3%)	12 (17.9%)	
6 - 10	11 (16.4%)	17 (25.4%)	
11 - 15	15 (22.4%)	16 (23.9%)	Z = -3.771, p < 0.001
16 - 20	6 (9.0%)	5 (7.5%)	
Over 20	14 (20.9%)	17 (25.4%)	

Percentages refer to valid responses only. The pair of responses would be excluded in the analysis if either the response to pre- or post-course is missing. *No pre-specified percentages for selection in this question. Respondents were allowed to write the percentages themselves. These percentages were grouped as ranges here.

**Table 4:** Changes in confidence and attitude in the care of patients with dermatological problems

	Pre-course	Post-course	Wilcoxon signed rank test
I am confident of *diagnosing *patients with common dermatological problems (e.g. eczema, allergy, drug eruptions, cutaneous infections) (n = 68)			
Strongly disagree	0 (0.0%)	0 (0.0%)	
Disagree	26 (38.2%)	6 (8.8%)	Z = -5.444, p < 0.001
Agree	39 (57.4%)	44 (64.7%)	
Strongly agree	3 (4.4%)	18 (26.5%)	
I am confident of *managing *common dermatological problems (n = 69)			
Strongly disagree	0 (0.0%)	1 (1.4%)	
Disagree	29 (42.0%)	7 (10.1%)	Z = -4.545, p < 0.001
Agree	38 (55.1%)	52 (75.4%)	
Strongly agree	2 (2.9%)	9 (13.0%)	
I am confident of *diagnosing *malignant skin tumours (n = 68)			
Strongly disagree	5 (7.4%)	1 (1.5%)	
Disagree	32 (47.1%)	18 (26.5%)	Z = -4.508, p < 0.001
Agree	29 (42.6%)	42 (61.8%)	
Strongly agree	2 (2.9%)	7 (10.3%)	
I am interested in following up patients with chronic dermatological problems (n = 68)			
Strongly disagree	2 (2.9%)	2 (2.9%)	
Disagree	25 (36.8%)	13 (19.1%)	Z = -3.962, p < 0.001
Agree	36 (52.9%)	41 (60.3%)	
Strongly agree	5 (7.4%)	12 (17.6%)	

Percentages refer to valid responses only. The pair of responses would be excluded in the analysis if either the response to pre- or post-course is missing.

The average percentage of patients with dermatological problems being referred to dermatologists reported by the respondents had dropped significantly from 31.9% before the course to 23.5% after completing the Course. Most patients were not referred at first consultation. Of all the referrals to dermatologists, the reported average percentage of patients being referred at first consultation decreased from 34.1% to 24.6%. Besides, the number of patients with dermatological problems seen by the respondents per day showed a significant increment after taking the Course.

Before taking the Course, less than two-third of the respondents were confident of diagnosing (61.8%) and managing (58.0%) common dermatological problems such as eczema, allergy, drug eruptions, cutaneous infections. After taking the Course, most were confident of diagnosing (91.2%) and managing (88.4%) these problems. A cross tabulation of the pre- and post-Course responses detailed the changes in the confidence level of the respondents in diagnosing or managing common dermatological problems (Table 5). The Kappa statistics and the p-values based on Bowker's test of symmetry for the confidence of diagnosing and managing common dermatological problems were 0.19, p < 0.001 and 0.22, p < 0.001 indicating that the disparity between the responses before and after taking the Course was quite large. Upon close scrutiny, a large proportion of respondents who were not that confident before the Course became confident or very confident after the Course. Furthermore, we particularly asked if the graduates were confident of diagnosing malignant skin tumours. The proportion of respondents rated themselves confident increased from 45.5% to 72.1% after taking the Course.

**Table 5:** A cross tabulation of the pre- and post-Course responses regarding confidence in diagnosing and managing patients with common dermatological problems

		Post-Course				
		Strongly disagree	Disagree	Agree	Strongly agree	Total
I am confident of diagnosing patients with common dermatological problems						
	Strongly disagree	0 (0.0%)	0 (0.0%)	0 (0.0%)	0 (0.0%)	0 (100.0%)
Pre-Course	Disagree	0 (0.0%)	6 (23.1%)	17 (65.4%)	3 (11.5%)	26 (100.0%)
	Agree	0 (0.0%)	0 (0.0%)	27 (69.2%)	12 (30.8%)	39 (100.0%)
	Strongly Agree	0 (0.0%)	0 (0.0%)	0 (0.0%)	3 (100.0%)	3 (100.0%)
	Total	0 (0.0%)	6 (8.8%)	44 (64.7%)	18 (26.5%)	68 (100.0%)
I am confident of managing common dermatological problems						
	Strongly disagree	0 (0.0%)	0 (0.0%)	0 (0.0%)	0 (0.0%)	0 (100.0%)
Pre-Course	Disagree	0 (0.0%)	7 (24.1%)	21 (72.4%)	1 (3.4%)	29 (100.0%)
	Agree	1 (2.6%)	0 (0.0%)	31 (81.6%)	6 (15.8%)	38 (100.0%)
	Strongly Agree	0 (0.0%)	0 (0.0%)	0 (0.0%)	2 (100.0%)	2 (100.0%)
	Total	1 (1.4%)	7 (10.1%)	52 (75.4%)	9 (13.0%)	69 (100.0%)

Graduates were also asked if they were interested in following up chronic dermatological problems. The combined percentage of "agree" and "strongly agree" increased from 60.3% to 77.9% after taking the Course.

### Qualitative Responses

Open-ended questions were designed to study the impact of the Course on the graduates and the barriers to implement what they learned. Most responses reflected on the increased confidence or knowledge, for example:

"Learn the updated management protocol/methods on common dermatological problems."

"It helps me to differentiate common dermatological illness, and not to miss important signs and symptoms."

Some graduates mentioned that they had increased understanding of their limitations. Some were also inspired to have further studies.

"Increase confidence in common diagnoses, know more clearly my limitations."

"Realise how much more knowledge I should learn."

"I now realise that undergraduate dermatology training was "no" training."

"Develop more interest in Dermatology."

Nonetheless, a few graduates mentioned their challenges in convincing patients for further investigations and lack of confidence and experience in some specific areas.

"Difficult to convince patient to have skin scrapping for investigation."

"To persuade patient to do further investigations is difficult, because most of the laboratory fee is expensive."

"I'm still not confident of skin biopsies."

## Discussion

We know of very few medical schools around the world that have developed similar postgraduate training programme to improve the dermatological knowledge and skills of primary care doctors. Less than one third of primary care doctors in the UK were reported to have received any postgraduate training in dermatology [11]. Our Course is the result of close collaboration between dermatologists and family physicians which has produced encouraging outcomes. The proportion of respondents being confident of diagnosing and managing common dermatological problems such as eczema, allergy, drug eruptions, cutaneous infections increased from about 60% to 90% after taking the Course.

Before taking the Course, the respondents would refer on average 31.9% of their patients with dermatological problems to dermatologists, which was slightly lower than the 37.5% reported in a US study [2]. The average referral percentage dropped significantly to 23.5% after taking the Course. Besides, there was an increase in the percentage of graduates being interested in following up patients with chronic dermatological problems. These results showed that our graduates were more ready to look after patients with common dermatological problems. This enhances the gate-keeping function of reducing unnecessary referrals to specialist dermatological services, especially in the public sector. These specialist dermatology clinics will also benefit from being able to concentrate their service to those difficult and complex cases requiring specialist care [20]. It is a win-win outcome for all parties concerned.

In recent years, the importance of early diagnosis of malignant skin tumour in primary care is emphasized [21]. Our study found that the proportion of graduates being confident of diagnosing malignant skin tumours increased from 45.5% to 72.1% after taking the Course. These changes suggest that our graduates are likely to be more effective in looking after patients with malignant skin tumours. These are in line with the findings by Westerhoff et al that Australian primary care doctors benefited from a training course in melanoma diagnosis using skin surface microscopy [22].

The improvements in knowledge and skills of primary care doctors towards dermatology are also important for the training of future doctors. Medical students are getting increasing curriculum time in primary care setting in many medical schools, where they can be potentially exposed to a wide variety of dermatological problems [9]. These learning opportunities can only be fully utilized if their primary care teachers are also competent in the field of dermatology.

This study has certain limitations. The self-reported questionnaire survey has attained a response rate of 42.9%, which though is not particularly high for this kind of study, but is already much better than most other surveys among doctors in Hong Kong [23-25]. It is possible that the respondents consist of a higher proportion of graduates who benefited relatively more (or less) from the Course. Moreover, the findings of this study came from doctors in the same healthcare system in Hong Kong which is a unique mix of private and public practice. Their educational and clinical needs may be different from doctors working in other countries. Despite that, the experience that we have gained from the present study should be useful to postgraduate medical educators in other countries in meeting the demand of healthcare services for patients with dermatological problems.

## Conclusions

The self-reported data from the graduates show that the Course is effective in improving their confidence and attitudes in looking after patients with common dermatological problems. There are significant changes in the reported practice characteristics of the graduates before and after taking the Course, including increased number of patients handled in relation to dermatological problems as well as decreased percentage of patients being referred.

## Abbreviations

CCCD: Certificate Course in Clinical Dermatology

## Competing interests

The authors declare that they have no competing interests.

## Authors' contributions

TPL, CKY and KFL designed the study. TPL and CKY managed the literature searches and wrote the protocol. TPL and CKY coordinated the study. KFL undertook the statistical analysis, and TPL managed the qualitative analysis. TPL, CKY and KFL wrote the first draft of the manuscript. All authors contributed to and have approved the final manuscript.

## Pre-publication history

The pre-publication history for this paper can be accessed here:

http://www.biomedcentral.com/1472-6920/11/20/prepub
